# High lncRNA H19 expression as prognostic indicator: data mining in female cancers and polling analysis in non-female cancers

**DOI:** 10.18632/oncotarget.13768

**Published:** 2016-12-01

**Authors:** Li Peng, Xiao-Qing Yuan, Zhao-Yang Liu, Wen-Ling Li, Chao-Yang Zhang, Ya-Qin Zhang, Xi Pan, Jun Chen, Yue-Hui Li, Guan-Cheng Li

**Affiliations:** ^1^ Key Laboratory of Carcinogenesis of the Chinese Ministry of Health and the Key Laboratory of Carcinogenesis and Cancer Invasion of Chinese Ministry of Education, Xiangya Hospital, Central South University, Changsha 410078, P.R. China; ^2^ Cancer Research Institute, Central South University, Changsha 410078, P.R. China; ^3^ Department of Clinical Pharmacology, Xiangya Hospital, Central South University, Changsha 410008, P.R. China; ^4^ Institute of Clinical Pharmacology, Central South University, Hunan Key Laboratory of Pharmacogenetics, Changsha 410078, P.R. China; ^5^ Department of Oncology, The third Xiangya Hospital, Central South University, Changsha 410013, P.R. China

**Keywords:** H19, prognosis, female cancers, TCGA, meta-analysis

## Abstract

Upregulation of lncRNA H19 expression is associated with an unfavorable prognosis in some cancers. However, the prognostic value of H19 in female-specific cancers has remained uncharacterized. In this study, the prognostic power of high H19 expression in female cancer patients from the TCGA datasets was analyzed using Kaplan-Meier survival curves and Cox's proportional hazard modeling. In addition, in a meta-analysis of non-female cancer patients from TCGA datasets and 12 independent studies, hazard ratios (HRs) with 95% confidence interval (CI) for overall survival (OS) and disease-free survival (DFS)/relapse-free survival (RFS)/metastasis-free survival (MFS)/progression-free survival (PFS) were pooled to assess the prognostic value of high H19 expression. Kaplan-Meier analysis revealed that patients with uterine corpus cancer and higher H19 expression had a shorter OS (HR=2.710, *p*<0.05), while females with cervical cancer and increased H19 expression had a shorter RFS (HR=2.261, *p*<0.05). Multivariate Cox regression analysis showed that high H19 expression could independently predict a poorer prognosis in cervical cancer patients (HR=4.099, *p*<0.05). In the meta-analysis, patients with high H19 expression showed a poorer outcome in non-female cancer (*p*<0.05). These results suggest that high lncRNA H19 expression is predictive of an unfavorable prognosis in two female cancers (uterine corpus endometrioid cancer and cervical cancer) as well as in non-female cancer patients.

## INTRODUCTION

Long non-coding RNAs (lncRNAs) are regulators of many important biological processes [1, 2], including cell growth, survival, migration, invasion, and differentiation [3–5]. H19 is a highly abundant, conserved, and imprinted lncRNA [6] that is overexpressed in several cancers and acts as an oncogene [7], promoting tumorigenesis and cancer progression [8, 9]. The upregulation of H19 forebodes an unfavorable prognosis in lung cancer [10], gastric cancer [11, 12], renal cell carcinoma [13], colorectal cancer [14], and gallbladder carcinoma [15]. However, the clinical significance and prognostic value of H19 in many other human tumors and female cancers, in particular, has remained uncharacterized.

Female cancers (breast, uterine, cervical, and ovarian cancer) are common malignant tumors [16], representing 2.714 million new cases (19.6 % of cases in both sexes and 41.5 % in women) and 1.016 million deaths worldwide (12.4 % death in both sexes and 28.6 % in women) according to the GLOBOCAN series of the International Agency for Research on Cancer [17]. The ratio of mortality to incidence is 32.5 % [17], suggesting a relatively poor prognosis for female cancers. Therefore, in order to improve the prognosis of female cancers, there is an urgent need to identify novel prognostic biomarkers.

In this study, we extracted data from five studies on female cancers (uterine corpus endometrioid cancer, cervical cancer, uterine carcinosarcoma, breast cancer, and ovarian cancer) and one study on all cancers (Pan-Cancer) from The Cancer Genome Atlas (TCGA). We evaluated the prognostic power of high lncRNA H19 expression for female cancers and pooled the prognostic ability of high H19 expression in non-female cancers according to TCGA datasets and the available literature. Our findings provide novel insights into prognostic indicators for corpus uteri and cervical cancer, and promote the clinical utility of H19 for individualized cancer treatments.

## RESULTS

### Patient characteristics from TCGA

Six independent datasets composed of TCGA Endometrioid Cancer (n=199), Cervical Cancer (n=308), Uterine Carcinosarcoma (n=57), Breast Invasive Carcinoma (n=1215), and Ovarian Cancer (n=266 and n=419), as well as one dataset of Pan-Cancer (n = 9755) were analyzed in May 2016. All patients with female cancers were female, except for 12 males who had breast cancer. Patient characteristics, including primary disease, RNAseq platform, number of patients, gender, age, TNM stage, tumor grade, tumor position, tumor residual disease (no macroscopic disease, 1-10 mm, 11-20 mm, >20 mm), lymphatic invasion, and venous invasion are shown in Table 1. There were no significant differences in clinicopathological variables between patients with high H19 expression and those with low H19 expression in patients with uterine corpus endometrioid cancer, cervical cancer, uterine carcinosarcoma, breast cancer, and ovarian cancer (*p* > 0.05; Supplementary Table S1–S6).

**Table 1:** Basic and clinic characteristics of cancer patients in 7 cohorts from TCGA database

Cohort	Pan- Cancer	Breast cancer	Ovarian cancer		Uterus cancer		
Primary disease	cancers	breast invasive carcinoma	ovarian serous cystadenocarcinoma		uterine carcinosarcoma	uterine corpus endometrioid carcinoma	cervical & endocervical adenocarcinoma
platform of RNAseq	Illumina HiSeq	IlluminaHiSeq, pancan normalized	IlluminaHiSeq, pancan normalized	Illumina Hiseq	Illumina HiSeq, pancan normalized	Illumina HiSeq, pancan normalized	Illumina HiSeq, pancan normalized
No. of patients	9755	1215	266	419	57	199	308
Gender							
Female	4675	1184	266	419	57	199	308
Male	4422	12	0	0	0	0	0
Age (years)							
<60	4357	647	149	223	6	50	240
≥60	4359	549	117	196	51	127	65
Clinical stage							
Tis	—	1	0	0	0	0	0
I	—	204	0	0	22	97	160
II	—	679	19	23	5	25	71
III	—	271	213	331	20	45	46
IV	—	21	33	62	10	10	21
X	—	18	0	0	0	0	0
T (tumor)							
Tis	—	0	—	—	—	—	1
1	—	311	—	—	—	—	139
2	—	691	—	—	—	73	—
3	—	143	—	—	—	—	21
4	—	47	—	—	—	—	9
X	—	4	—	—	—	—	17
N (Node)							
0	—	553	—	—	—	—	135
1	—	410	—	—	—	—	60
2	—	129	—	—	—	—	0
3	—	81	—	—	—	—	0
X	—	23	—	—	—	—	65
M (Metastasis)							
0	—	1010	—	—	—	—	115
1	—	22	—	—	—	—	10
X	—	164	—	—	—	—	130
Grade							
G1	—		1	—	—	13	20
G2	—		33	—	—	20	136
G3	—		225	—	—	140	118
G4	—		1	—	—	4	1
GB	—		1	—	—	0	0
GX	—		3	—	—	0	23
Position							
Left	—	625	34	56	—	—	—
Right	—	571	34	46	—	—	—
Bilateral	—		185	292	—	—	—
Tumor residual disease							
No Macroscopic disease	—	—	54	76	—	—	—
1-10 mm			119	196			
11-20 mm	—	—	20	29	—	—	—
>20 mm		—	42	76	—	—	—
Lymphatic invasion							
Positive	—	—	70	106	—	—	—
Negative	—	—	39	55	—	—	—
Venous invasion							
Positive	—	—	44	65	—	—	—
Negative	—	—	38	48	—	—	—

### High H19 expression predicts a shorter median overall survival in uterine corpus endometrioid cancer patients

**Figure 1:**
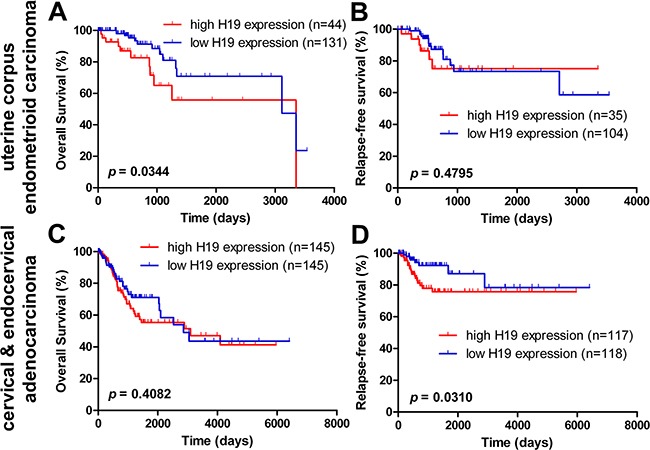
Kaplan-Meier estimate of overall survival and relapse-free survival of H19 expression in endometrioid carcinoma and cervical cancer patients The overall survival (OS) **A.** and relapse-free survival (RFS) **B.** of H19 expression in uterine corpus cancer patients from TCGA dataset (Uterine Corpus Endometrioid Carcinoma – IlluminaHiSeq - pancan normalized; n _OS_ = 175 and n _RFS_ = 139). The OS **C.** and RFS **D.** of H19 expression in cervical cancer patients from the TCGA dataset (Cervical & Endocervical Adenocarcinoma - IlluminaHiSeq - pancan normalized and Uterine Corpus Endometrioid Carcinoma – IlluminaHiSeq - pancan normalized, n _OS_ = 290 and n _RFS_ = 235).

We first evaluated the prognostic power of H19 in five female cancers. Patients with uterine corpus endometrioid cancer from the TCGA Endometrioid Cancer cohort (gene expression by RNAseq -- IlluminaHiSeq, pancan normalized, n=199) who had high H19 expression had unfavorable overall survival (OS; HR = 2.710, 95% CI = 1.076 - 6.827, *p* = 0.0344), while there was no effect on relapse-free survival (RFS) time (*p* > 0.05) in comparison with those uterine corpus endometrioid cancer patients with low H19 expression (Figure 1A-1B).

Univariate and multivariate Cox's proportional hazards analyses were conducted to determine the prognostic value of H19 in the OS of uterine corpus endometrioid cancer patients. In the univariate Cox regression analysis, higher H19 expression (HR = 2.281, 95% CI = 1.020 - 5.099, *p* = 0.045), heavier weight (HR = 0.385, 95% CI = 0.148 - 1.002, *p* = 0.050), larger BMI (HR = 0.275, 95% CI = 0.100 - 0.757, *p* = 0.012), and higher clinical TNM stage (HR = 3.526, 95% CI = 1.479 - 8.404, *p* = 0.004) were all associated with uterine corpus endometrioid carcinoma patients' OS (Table 2). Multivariate Cox regression revealed that high H19 expression had no effect on OS in uterine corpus endometrioid cancer patients (Table 2). Yet, there was a prognostic impact of larger BMI (HR = 0.173, 95% CI = 0.037 - 0.809, *p* = 0.026) and higher clinical TNM stage (HR = 4.834, 95% CI = 1.495 – 15.630, *p* = 0.009) on OS (Table 2). Taken together, these results indicated that H19 predicts a shorter OS in uterine corpus endometrioid cancer patients.

**Table 2:** Univariate and multivariate analysis of clinic pathologic factors for overall survival of 199 uterine corpus endometrioid carcinoma patients

Risk factors	Univariate analysis			Multivariate analysis		
	HR	95 % CI	*p*	HR	95 % CI	*p*
H19 expression (high, vs. low)	2.281	1.020-5.099	0.045	1.223	0.445-3.366	0.634
Age (≥ 60, vs. < 60)	1.362	0.562-3.298	0.494			
Weight (≥ 80, vs. < 80)	0.385	0.148-1.002	0.050	1.781	0.226-2.347	0.667
Height (≥ 161, vs. < 161)	1.026	0.450-2.335	0.952			
BMI (≥25, vs. <25)	0.037	0.000-23.998	0.318			
BMI (≥15.625, vs. <15.625)	0.275	0.100-0.757	0.012	0.173	0.037-0.809	0.026
Clinical stage (III- IV, vs. I-II)	3.526	1.479-8.404	0.004	4.834	1.495-15.630	0.009
Grade (3-4, vs. 1-2)	26.927	0.192-3373.954	0.192			
Sample type (non-, vs. solid tissue normal)	1.608	0.368-7.028	0.528			
Colorectal cancer (positive, vs. negative)	0.049	0.000-2.05E11	0.839			
Diabetes (positive, vs. negative)	0.314	0.072-1.368	0.123			
Hypertension (positive, vs. negative)	0.403	0.146-1.114	0.080	0.775	0.256-2.347	0.792
Pregnancies (>2, vs.≤2)	1.663	0.618-4.478	0.314			
Histological type (mix, vs. serous/endometrioid)	3.095	0.894-10.717	0.075	2.117	0.568-7.890	0.330
Menopause status (Post, vs. Peri/Pre)	2.160	0.289-16.118	0.453			

### High H19 expression is an independent prognostic factor for cervical cancer patients' relapse-free survival

In cervical & endocervical cancer patients from the TCGA Cervical Cancer cohort (gene expression by RNAseq -- IlluminaHiSeq, pancan normalized, n=308), high H19 expression did not predict OS (*p* > 0.05). However, high H19 expression was associated with a shorter relapse-free survival (RFS; HR = 2.261, 95% CI = 1.077 - 4.747, *p* = 0.0310) in cervical cancer patients when compared with patients with low H19 expression (Figure 1C-1D).

**Figure 2:**
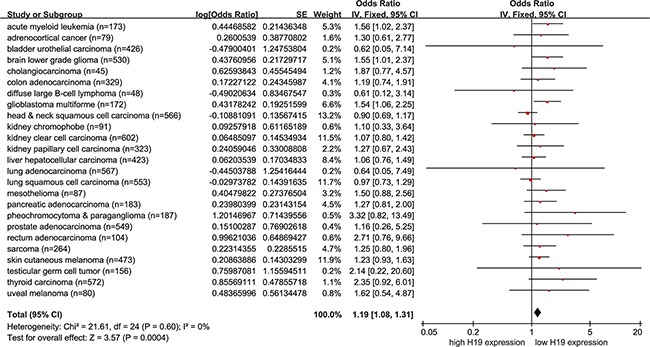
Pooling analysis estimate of overall survival of H19 expression in non-female cancer patients from the pan-cancer cohort The overall survival (OS) of H19 expression in non-female cancer patients from the Kaplan-Meier analaysis of the Pan-Cancer dataset. The size of the blocks or diamonds represents the weight of the random-effect model in the meta-analysis. HR > 1 indicates that high H19 expression is correlated with a more unfavorable OS.

Univariate and multivariate Cox's proportional hazards analyses were conducted to determine the prognostic value of H19 on RFS of cervical cancer patients. In the univariate Cox regression analysis, there was a prognostic influence of higher H19 expression (HR = 2.397, 95% CI = 1.055 - 5.442, *p* = 0.037), higher Node (N) phase of TNM stage (HR = 3.759, 95% CI = 1.535 - 9.203, *p* = 0.004), and tobacco smoking (HR = 0.405, 95% CI = 0.170 - 0.968, *p* = 0.042) on RFS in cervical cancer patients (Table 3). Multivariate Cox regression further verified the prognostic value of higher H19 expression (HR = 4.099, 95% CI = 1.156 - 14.538, *p* = 0.029) and higher Node (N) phase of TNM stage (HR = 4.186, 95% CI = 1.614 - 10.856, *p* = 0.003) served as independent prognostic predictors for RFS in cervical cancer patients. In sum, these results indicated that H19 serves as a prognostic indicator of RFS in cervical cancer patients (Table 3).

**Table 3:** Univariate and multivariate analysis of clinic pathologic factors for relapse-free survival of 308 cervical & endocervical adenocarcinoma patients

Risk factors	Univariate analysis			Multivariate analysis		
	HR	95 % CI	*p*	HR	95 % CI	*p*
H19 expression (high, vs. low)	2.397	1.055-5.442	0.037	4.099	1.156-14.538	0.029
Age (≥ 60, vs. < 60)	0.938	0.380-2.316	0.890			
Weight (≥ 71, vs. < 71)	0.871	0.402-1.885	0.725			
Height (≥ 161, vs. < 161)	0.720	0.298-1.739	0.465			
BMI (≥ 25, vs. < 25)	0.049	0.000-1.266E50	0.960			
BMI (> 13.304, vs. < 13.304)	0.548	0.227-1.324	0.181			
Clinical stage (III- IV, vs. I-II)	0.505	0.152-1.674	0.264			
T (Tumor; 3-4, vs. 1-2)	0.768	0.180-3.283	0.722			
N (Node; 1, vs. 0)	3.759	1.535-9.203	0.004	4.186	1.614-10.856	0.003
M (Metastasis; 1, vs. 0)	0.045	0.000-2410.453	0.577			
Grade (3, vs. 1-2)	1.554	0.740-3.263	0.244			
Sample type (non-, vs. solid tissue normal)	20.622	0.000-3.065E7	0.676			
Pregnancies (>2, vs.≦2)	0.957	0.361-2.539	0.929			
Ectopic pregnancies (>1, vs. 0)	0.041	0.000-51.985	0.381			
Pregnancy spontaneous abortion (>1, vs. 0)	0.804	0.279-2.317	0.686			
Tobacco smoking (yes, vs. no)	0.405	0.170-0.968	0.042	0.342	0.096-1.211	0.096
Tobacco smoking (current, vs. non/reformed)	0.456	0.157-1.327	0.149			
Histological type (Squamous, vs. others)	1.376	0.477-3.969	0.554			
HPV (HPV16/18, vs. others)	1.172	0.106-12.978	0.897			
Menopause status (Post, vs. Peri/Pre)	0.516	0.205-1.301	0.161			

### Prognostic power of high H19 expression in other female cancer patients

We also evaluated the prognostic ability of high H19 expression in three additional female cancer subtypes including uterine carcinosarcoma (n=57), breast cancer (n=1215), and ovarian cancer (n=266 and n=419) from the TCGA datasets. There were no significant differences on OS or RFS between patients with higher H19 expression and those with lower H19 expression in these three cancers (*p* > 0.05, Supplementary Figure S2A-S2F).

### Prognostic analysis of high H19 expression in non-female cancer patients from the TCGA cohorts

Because the above results suggested that high H19 expression could predict an unfavorable prognosis in some female cancer patients, we next assessed whether it had prognostic ability in patients with non-female cancer (Pan-cancer excluding the female cancers, including 25 cancers). As there are large differences in mean H19 expression among different kinds of tumors (data not shown), the HRs with 95% CI of OS and RFS from Kaplan-Meier analysis for non-female cancers of TCGA cohorts were pooled to assess the prognostic value of high H19 expression. A shorter OS (pooling HR = 1.19, 95% CI = 1.08 - 1.31, *p* = 0.0004) was observed in non-female cancer patients with higher H19 expression compared with those with lower H19 expression under a fixed-effect model (Figure 2, Supplementary Figure S3). Yet, there were no differences in RFS between the groups of non-female cancer patients with higher H19 expression and those with lower H19 expression with a fixed-effect model (*p* > 0.05, Supplementary Figure S4-S5). It is worth noting that there was no significant heterogeneity (*p*' = 0.60, *I^2^* = 0 %; *p*' = 0.72, *I^2^* = 0 %) in either OS or RFS (Supplementary Figure S6).

### Characteristics of publications in meta-analysis

To further validate the prognostic value of high H19 expression in cancer patients, twelve studies [11–15, 18–24] totaling 878 individuals were incorporated into a meta-analysis. The clinicopathological characteristics of these patients are shown in Table 4. Sample sizes ranged from 24 to 128 patients. Three studies were designed for hepatic cancer, three for gastric cancer, one for non-small cell lung cancer, one for colorectal cancer, one for clear cell renal cell carcinoma, one for bladder cancer, one for gallbladder cancer, and one for glioblastoma. *H19* expression was detected in two studies by ISH and in ten by RT-PCR (Table 4).

**Table 4:** Characteristics of the eligible studies in meta-analysis

First author	Year	Region	Age	No of patients	Sex (M/F)	Cancer type	Tumor stage	Detection method	Survival analysis	Outcomes	Follow-up, months
I Ariel	2000	Israel	68 (55-81)	61	48/12	Bladder cancer	0-IV	ISH	Univariate	DFS	—
N Iizuka	2004	Yamaguchi		59		HCC	I-III	RT-PCR	Univariate	RFS	—
Y Fellig	2005	Israel	60.8 (13.3-80.0)	64	45/35	Hepatic metastases	—	ISH	Univariate	MFS; OS	—
L Zhang	2013	China	—	113	—	HCC	—	qRT-PCR	Univariate	DFS	21 (1-24)
EB Zhang	2014	China	—	80	47/33	Gastric cancer	I-IV	qRT-PCR	Univariate and Multivariate	OS	—
H Li	2014	China	—	74	54/20	Gastric cancer	I-IV	qRT-PCR	Univariate	OS	—
L Wang	2015	China	—	92	57/35	ccRCC	I-IV	qRT-PCR	Univariate and Multivariate	OS	—
EB Zhang	2015	China	—	70	46/24	NSCLC	I-IV	qRT-PCR	Univariate and Multivariate	OS	—
XC Jiang	2016	China	—	30	—	Glioblastoma	—	qRT-PCR	Univariate	PFS	—
SH Wang	2016	China	—	24	6/18	Gallbladder cancer	—	qRT-PCR	Univariate	OS	—
JS Chen	2016	China	—	128	79/49	Gastric cancer	—	qRT-PCR	Univariate and Multivariate	DFS; OS	36 (20-48)
D Han	2016	China	—	83	40/43	Colorectal cancer	I-IV	qRT-PCR	Univariate and Multivariate	DFS; OS	—

M/F, Male/Female; DFS, disease-free survival; HCC, Hepatocellular Carcinoma; ccRCC, Clear Cell Renal Cell Carcinoma; NSCLC, Non Small Cell Lung Cancer; ISH, in situ hybridization; qRT-PCR, quantitative real-time polymerase chain reaction; RFS, relapse-free survival; OS, overall survival; MFS, metastasis-free survival; PFS, progression-free survival; —, there is no corresponding data presented.

### A meta-analysis to assess the prognostic power of H19 in non-female cancers

**Figure 3:**
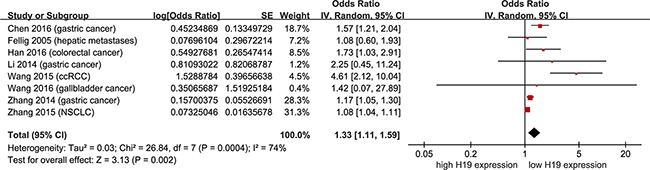
Meta-analysis of HRs with 95%CI for overall survival from the univariate analysis of cancers from the available literature The size of the blocks or diamonds represents the weight for the random-effect model in the meta-analysis. HR > 1 indicates that high H19 expression is correlated with a more unfavorable overall survival (OS).

**Figure 4:**
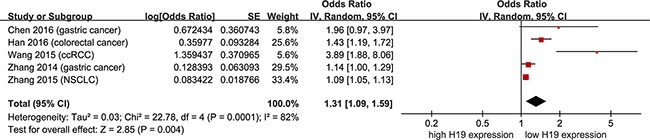
Meta-analysis of HRs with 95%CI for overall survival from the multivariate analysis of cancers from the available literature The size of the blocks or diamonds represents the weight for the random-effect model in the meta-analysis. HR > 1 indicates that high H19 expression is correlated with a more unfavorable overall survival (OS).

As shown in Figure 3, data were derived from univariate Cox analysis of 8 studies, totaling 615 non-female cancer patients. Under a random-effect model, non-female cancer patients with high H19 expression had shorter OS than those with low H19 expression (Figure 3; pooling HR = 1.33, 95 % CI = 1.11 - 1.59, *p* = 0.002). In addition, data were derived from multivariate Cox analysis of 5 studies, totaling 453 patients. With a random-effect model, shorter OS was observed in non-female cancer patients with high H19 expression than those with low H19 expression (Figure 4; pooling HR = 1.31, 95 % CI = 1.09 - 1.59, *p* = 0.004). These results suggested that high H19 expression could predict inferior clinical outcomes on OS time in non-female cancer patients.

**Figure 5:**
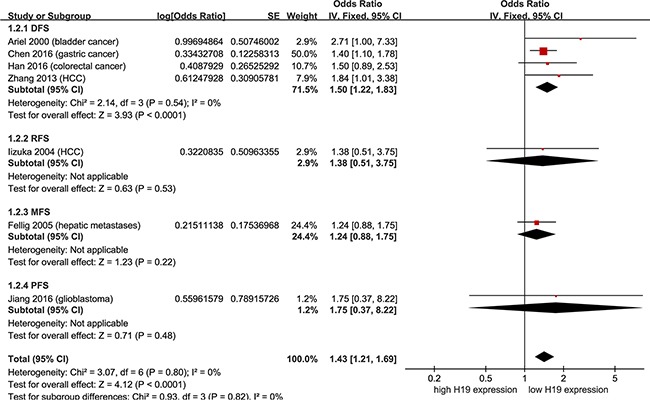
Meta-analysis of HRs with 95%CI for DFS/RFS/MFS/PFS from the univariate analysis of cancers from the available literature The size of the blocks or diamonds represents the weight for the random-effect model in the meta-analysis. HR > 1 indicates that high H19 expression is correlated with a more unfavorable disease-free survival (DFS), relapse-free survival (RFS), metastasis-free survival (MFS) and progression-free survival (PFS).

**Figure 6:**
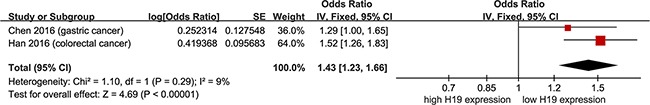
Meta-analysis of HRs with 95%CI for disease-free survival from the multivariate analysis of cancers from the available literature The size of the blocks or diamonds represents the weight for the random-effect model in the meta-analysis. HR > 1 indicates that high H19 expression is correlated with a more unfavorable disease-free survival (DFS).

To assess RFS, disease-free survival (DFS), metastasis-free survival (MFS), and progression-free survival (PFS), data were extracted from univariate Cox analysis of 7 studies, totaling 538 non-female cancer patients. As shown in Figure 5, there was no heterogeneity (*p*' = 0.54, *I^2^* = 0 %; *p*' = 0.80, *I^2^* = 0 %) in DFS or DFS/RFS/MFS/PFS. With a fixed-effect model, shorter DFS (pooling HR = 1.50, 95 % CI = 1.22 - 1.83, *p* < 0.0001) and DFS/RFS/MFS/PFS (pooling HR = 1.43, 95 % CI = 1.21 - 1.69, *p* = 0.004) were seen in non-female cancer patients with higher H19 expression in comparison to those with lower H19 expression (Figure 5). Data were then derived from multivariate Cox analysis of 2 studies, totaling 211 patients. With a fixed-effect model, non-female cancer patients with high H19 expression had a shorter DFS than those with low H19 expression (Figure 6; pooling HR = 1.43, 95 % CI = 1.23 - 1.66, *p* < 0.0001). These results suggested that high H19 expression could predict an adverse prognosis on DFS or DFS/RFS/MFS/PFS in non-female cancer patients, and predict adverse OS and DFS/RFS/MFS/PFS in non-female cancer patients by pooling meta-analysis.

An assessment of publication bias was conducted using RevMan. The funnel plots of DFS/RFS/MFS/PFS showed that the dots were symmetrically distributed, indicating that there was no remarkable bias, and thus, our results are credible and reliable. Although the shape of funnel plots in OS outcomes did not meet global symmetry, random-effect models were used and thus our results merit consideration. The results of funnel plot are shown in Supplementary Figure S7.

## DISCUSSION

Although there have been considerable advances in the development of therapies, the overall outcomes of patients with female cancers remain dismal [17]. Thus, it is necessary to discover new prognostic biomarkers and therapeutic targets. This study found that lncRNA H19 expression acts as a novel prognostic factor for uterine corpus endometrioid cancer and cervical cancer, as well as a prognostic indicator of non-female cancers. LncRNA H19, one of the first imprinted genes to be identified, is expressed in both embryonic and extra-embryonic cell lineages [25]. H19 is located at chromosome 11p15.5 encoding a 2.3 kb lncRNA [26], is frequently deregulated in tumors, and contributes to both cancer initiation and progression [27]. Thus, H19 may play an indispensable role in the pathogenesis of cancers. In recent years, there have been a growing number of reports on the biological functions of H19 in cancers [8, 28–33], but few studies on the prognostic significance of H19 expression in cancers, especially female cancers. Therefore, we focused on the evaluation of lncRNA H19 as an outcome predictor in female cancers.

Female cancers, including cancers of the breast, uterus, ovary, and cervix, account for over 40% of newly diagnosed malignancies and for approximately 30% of malignancy-associated deaths in women worldwide [17], and have a relatively poor prognosis [34]. In order to assess the potential prognostic value of H19 expression in female cancers, we analyzed six datasets for female cancers from TCGA, and discovered that high expression of H19 had an unfavorable prognostic influence on OS and RFS in uterine corpus endometrioid cancer and cervical cancer, but not in breast cancer and ovarian cancer. Although there were no statistical differences between the two groups of higher and lower H19 expression in breast or ovarian cancer, there was an “HR >1” in almost all of our analyses of breast and ovarian cancer.

To understand the extensive role of H19 in cancers, we then implemented a meta-analysis of Kaplan-Meier survival analysis to evaluate the prognostic power of H19 in non-female cancers (25 common cancers) from TCGA. Consistent with some reports that H19 expression could serve as a poor prognostic factor [12–14, 18, 23, 24], our results further indicated that high expression of H19 predicted an unfavorable prognosis in non-female cancer patients. However, there is still controversy about whether H19 expression can predict the prognosis of certain cancers. Some studies showed negative findings on RFS/MFS and OS [19, 20]. Thus, it was necessary to carry out a meta-analysis to further elaborate the prognostic value of H19 expression based on the published literature. These meta-analysis results showed that high H19 expression may serve as a poor prognostic indicator in cancer patients which were in line with our pooling analysis based upon the TCGA database.

In conclusion, we found that high lncRNA H19 expression predicted an inferior prognosis in two female cancers (uterine corpus endometrioid cancer and cervical cancer), as well as in non-female cancer patients. We also propose a cost-efficient and effective way to identify prognostic biomarkers and even provide an insight for the predicted outcome of cancer patients. In the future, further studies with larger samples are required to ascertain the prognostic significance of H19 expression in cancers, especially in female cancers. Also, the biological functions and molecular mechanisms of lncRNA H19 in cervical cancer and uterine corpus endometrioid cancer are worthy of further research to furnish experimental evidence for its utility as a potential biomarker of disease prognosis and precision treatment.

## MATERIALS AND METHODS

### Data extracted from the TCGA database

Six independent datasets including the information on mRNA expression and clinical features of female cancers as well as one dataset on Pan-Cancer (n = 9755 by IlluminaHiSeq, 31 cancers with available expression and survival data) were obtained from the UCSC Cancer Genomics Browser of TCGA (https://genome-cancer.soe.ucsc.edu/). The six cohorts were composed of: one breast cancer (n=1215 for Breast Invasive Carcinoma by pancan normalized IlluminaHiSeq), three uterine cancer (n=57 for Uterine Carcinosarcoma, n=199 for Uterine Corpus Endometrioid Carcinoma and n=308 for Cervical Squamous Cell Carcinoma and Endocervical Adenocarcinoma, all by pancan normalized IlluminaHiSeq), and two ovarian cancer (n=266 and n=419 for Ovarian Serous Cystadenocarcinoma by pancan normalized IlluminaHiSeq and IlluminaHiseq, respectively). Then, we evaluated the association of lncRNA H19 expression with OS and/or RFS of three female cancers in the six cohorts. Furthermore, we extracted the expression and survival data of 25 non-female cancers (including acute myeloid leukemia, adrenocortical cancer, bladder urothelial carcinoma, brain lower grade glioma, cholangiocarcinoma, colon adenocarcinoma, diffuse large B-cell lymphoma, glioblastoma multiforme, head & neck squamous cell carcinoma, kidney chromophobe, kidney clear cell carcinoma, kidney papillary cell carcinoma, liver hepatocellular carcinoma, lung adenocarcinoma, lung squamous cell carcinoma, mesothelioma, pancreatic adenocarcinoma, pheochromocytoma & paraganglioma, prostate adenocarcinoma, rectum adenocarcinoma, sarcoma, skin cutaneous melanoma, testicular germ cell tumor, thyroid carcinoma and uveal melanoma) from the Pan-Cancer dataset. Also, patients were classified into two groups of higher H19 expression and lower H19 expression by median or upper quartile according to the published literature and distribution in terms of box plots on H19 expression in female cancer patients (Supplementary Figure S1). When the upper quartile was applied as a cut-off value, it indicated that higher H19 expression can only predict a significant prognosis. In addition, the associations between H19 expression and OS/RFS in these cohorts were also analyzed.

### Kaplan-Meier survival analysis and Cox regression analysis

Differences between the clinicopathological data of higher H19 expression and those of lower H19 expression were assessed using the Chi-squared test. For survival analysis, OS was assessed from the day of diagnosis to the day of last follow-up, while RFS was defined as the time from the day of the first complete remission to the day of first relapse or death [35–37]. Survival curves were established using the Kaplan-Meier approach, with log-rank tests was applied to appraise the differences between the groups. HRs were estimated using Cox's proportional hazards model. Univariate and multivariate Cox models for the prognostic effect of H19 expression on OS/RFS in female cancer patients from the TCGA were analyzed. SPSS 17.0 software (IBM, Chicago, USA) was used to conduct statistical analysis and a two-sided *p*-value < 0.05 was regarded as statistical significance. All survival-related figures were plotted in GraphPad Prism 5 (GraphPad, La Jolla, USA).

### Literature search for meta-analysis

Literature were retrieved from PubMed, Embase, Web of Science, ClinicalTrials and the Cochrane Library with the following search terms: ‘imprinted maternally expressed transcript’, ‘lncRNA H19’, ‘H19 RNA’, ‘H19’, ‘ASM’, ‘BWS’, ‘WT2’, ‘ASM1’, ‘D11S813E’, ‘LINC00008’, or ‘NCRNA00008’; AND ‘Neoplasm’, ‘Neoplasms’, ‘Tumor’, ‘Tumors’, ‘Neoplasia’, ‘Cancer’, ‘Cancers’, ‘malignancy’, ‘sarcoma’, ‘adenoma’, or ‘melanoma’; AND ‘prognostic’, ‘prognosis’, ‘outcome’, ‘outcomes’, ‘mortality’, ‘survival’, ‘overall survival’, ‘OS’, ‘disease-free survival’, ‘DFS’, ‘relapse-free survival’, ‘RFS’, ‘metastasis-free survival’, ‘MFS’, ‘progression-free survival’, or ‘PFS’.

### Study selection and data extraction for meta-analysis

No related review protocol has been published. Inclusion criteria: (1) studies were full papers in English prior to April 5, 2016; (2) studies were original cohort studies; (3) patients were grouped in terms of the expression of H19, (4) studies were focused on the prognostic effect of H19 on patients with any type of cancer; (5) data was available on survival including OS and/or DFS/RFS/MFS/PFS. Exclusion criteria: (1) Non-human research; (2) meta-analysis, letters, expert opinions, comments, case reports and reviews; (2) duplicate publications; (3) studies without qualified data; (5) not yet published in English. Repetitive literature was managed and removed by Endnote X7.

Two researchers independently inspected all the literature that satisfied the inclusion criteria, and divergences between reviewers were settled through discussion. Information, including first author, year of publication, study region, median age, sample size, sex distribution, cancer type, tumor stage, detection method, and follow-up times from each eligible study was extracted. The corresponding HRs with 95% CI for OS and DFS/RFS/MFS/PFS were calculated from COX univariate models from corresponding Kaplan-Meier curves by the methods [38, 39], as well as COX multivariate models.

### Statistics for meta-analysis

Meta-analysis was performed with the software of Review Manager (RevMan) (version 5.3.5; the Nordic Cochrane Centre, Copenhagen, Denmark). Prognostic roles of H19 expression on OS and/or DFS/RFS/MFS/PFS were assessed by estimation of the pooled HRs and their matching 95% CI with the inverse variance method. Statistical heterogeneity was assessed using the chi-squared test (the significance of heterogeneity was artificially expressed as *p'*-value to distinguish from the significance of outcomes) and *I^2^* statistics. When there was no significant heterogeneity (*p'*-value > 0.1 and *I^2^* < 50%), the pooled HRs were assessed by fixed-effect models. Otherwise, random-effect models were utilized to enhance the stability of the meta-analysis. Publication bias was appraised by Begg funnel plot and Egger's test.

## SUPPLEMENTARY FIGURES AND TABLES














