# Supplementary material for: High lncRNA H19 expression as prognostic indicator: data mining in female cancers and polling analysis in non-female cancers
Source: Oncotarget. 2016 Dec 1;8(1):1655–67. doi: 10.18632/oncotarget.13768 (PMC5352086; doi:10.18632/oncotarget.13768)
Supplement: Supplementary file 1 [file oncotarget-08-1655-s001.pdf]

High lncRNA H19 expression as prognostic indicator: data mining in female cancers and polling analysis in non-female cancers

SUPPLEMENTARY FIGURES AND TABLES

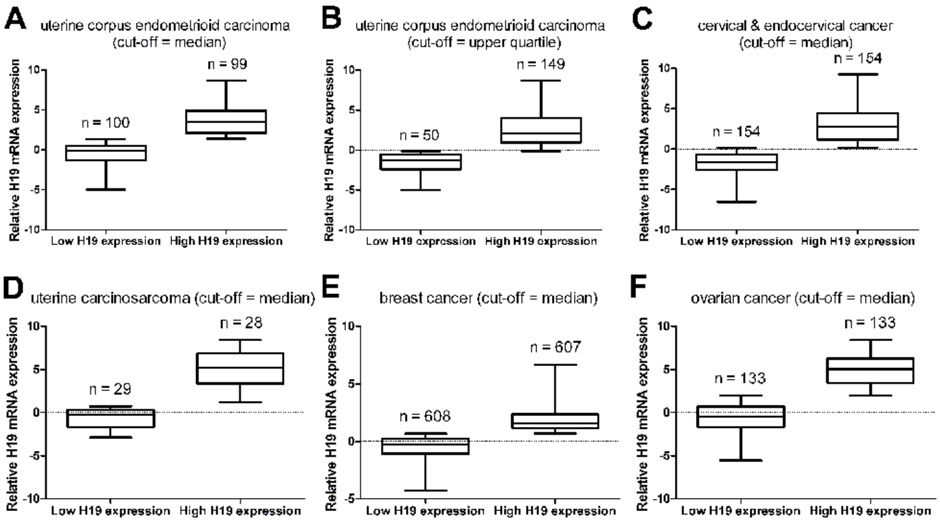

Supplementary Figure S1: Distributions in terms of box plots on H19 expression in five female cancers. The box plots of distributions of H19 expression in uterine corpus endometrioid cancer **A, B.** cervical cancer **C.** uterine carcinosarcoma **D.** breast cancer **E.** and ovarian cancer **F.**.

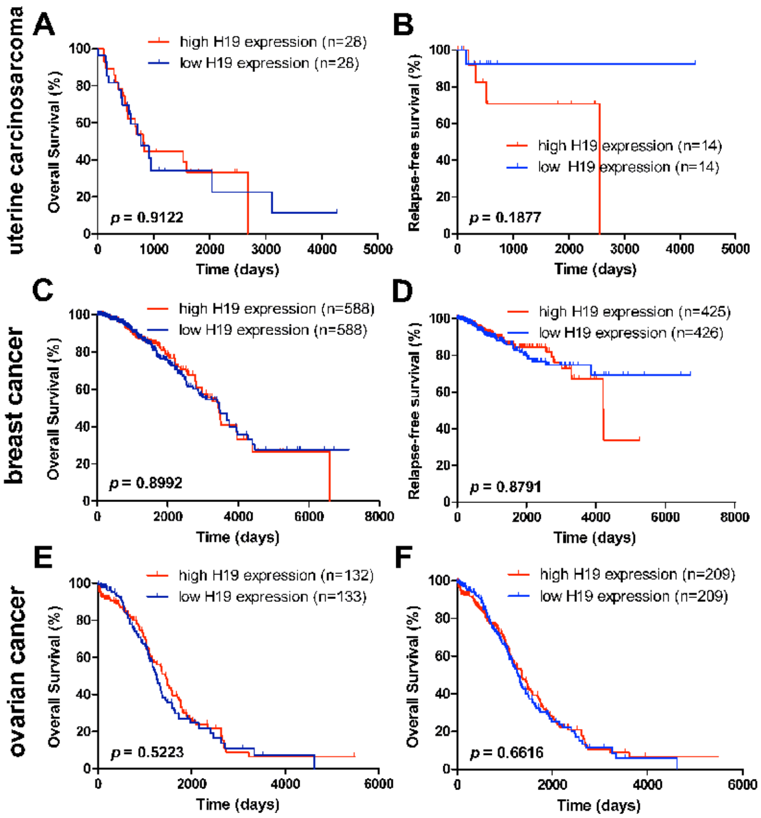

Supplementary Figure S2: Kaplan-Meier estimate for OS and RFS of H19 expression in some female cancer patients. The OS **A.** and RFS **B.** of H19 expression were shown in uterine carcinosarcoma patients from TCGA dataset (Uterine Carcinosarcoma -IlluminaHiSeq- pancan normalized; n_{OS} = 56 and n_{RFS} = 28). The OS **C.** and RFS **D.** of H19 expression in breast cancer patients from the TCGA dataset (Breast Invasive Carcinoma-IlluminaHiSeq- pancan normalized, (n_{OS} = 1176 and n_{RFS} = 851)). The OS **E, F.** of H19 expression in ovarian cancer patients from two different TCGA datasets (Ovarian Serous Cystadenocarcinoma – IlluminaHiSeq - pancan normalized and RNAseq - IlluminaHiseq BC, n_{OS} = 265 and 418).

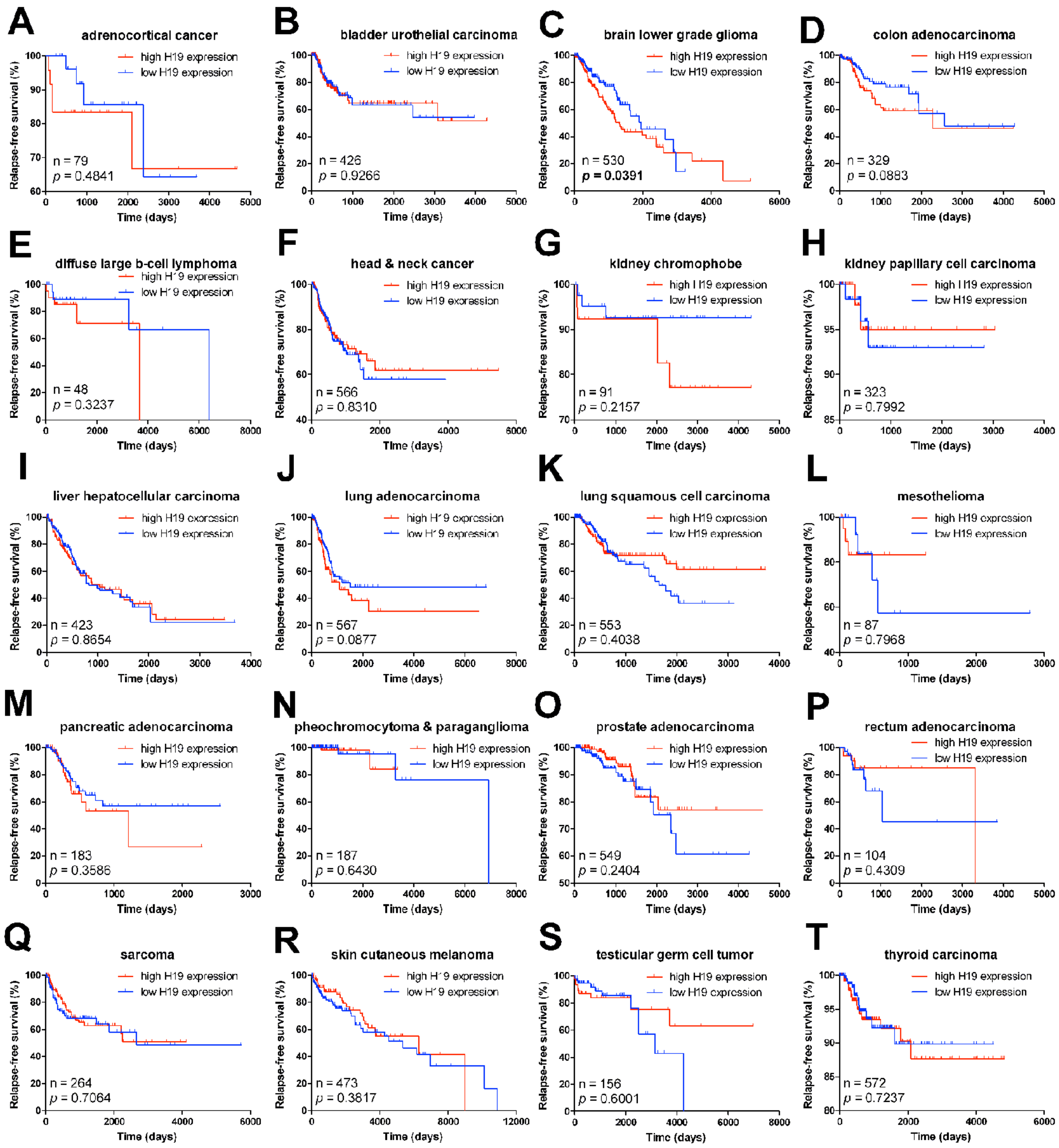

Supplementary Figure S3: Kaplan-Meier estimate for RFS of high H19 expression in non-female cancer patients. The relapse-free survival (RFS) of high H19 expression in adrenocortical cancer **A.** bladder urothelial carcinoma **B.** brain lower grade glioma **C.** colon adenocarcinoma **D.** diffuse large B-cell lymphoma **E.** head & neck squamous cell carcinoma **F.** kidney chromophobe **G.** kidney papillary cell carcinoma **H.** liver hepatocellular carcinoma **I.** lung adenocarcinoma **J.** lung squamous cell carcinoma **K.** mesothelioma **L.** pancreatic adenocarcinoma **M.** pheochromocytoma & paraganglioma **N.** prostate adenocarcinoma **O.** rectum adenocarcinoma **P.** sarcoma **Q.** skin cutaneous melanoma **R.** testicular germ cell tumor **S.** and thyroid carcinoma **T.** patients from TCGA datasets.

Study or Subgroup	log[Odds Ratio]	SE	Weight	Odds Ratio IV, Fixed, 95% CI
adrenocortical cancer (n=79)	0.47062843	0.67260716	1.1%	1.60 [0.43, 5.98]
bladder urothelial carcinoma (n=426)	0.02371653	0.25678088	7.8%	1.02 [0.62, 1.69]
brain lower grade glioma (n=530)	0.40812823	0.19756884	13.2%	1.50 [1.02, 2.22]
colon adenocarcinoma (n=329)	0.46624658	0.27355984	6.9%	1.59 [0.93, 2.72]
diffuse large B-cell lymphoma (n=48)	0.70902053	0.71829117	1.0%	2.03 [0.50, 8.30]
head & neck squamous cell carcinoma (n=566)	-0.04751098	0.22283659	10.4%	0.95 [0.62, 1.48]
kidney chromophobe (n=91)	0.83290912	0.67287917	1.1%	2.30 [0.62, 8.60]
kidney papillary cell carcinoma (n=323)	-0.22865873	0.89871308	0.6%	0.80 [0.14, 4.63]
liver hepatocellular carcinoma (n=423)	0.03149867	0.18692313	14.8%	1.03 [0.72, 1.49]
lung adenocarcinoma (n=567)	-0.32573014	0.87983752	0.7%	0.72 [0.13, 4.05]
lung squamous cell carcinoma (n=553)	-0.19394889	0.23232781	9.6%	0.82 [0.52, 1.30]
mesothelioma (n=87)	-0.19613655	0.76164937	0.9%	0.82 [0.18, 3.66]
pancreatic adenocarcinoma (n=183)	0.3074847	0.33519737	4.6%	1.36 [0.71, 2.62]
pheochromocytoma & paraganglioma (n=187)	0.4916428	1.06397699	0.5%	1.63 [0.20, 13.16]
prostate adenocarcinoma (n=549)	-0.41188568	0.35068086	4.2%	0.66 [0.33, 1.32]
rectum adenocarcinoma (n=104)	-0.44021184	0.55897148	1.6%	0.64 [0.22, 1.93]
sarcoma (n=264)	-0.10280822	0.27283033	6.9%	0.90 [0.53, 1.54]
skin cutaneous melanoma (n=473)	-0.21877809	0.2501597	8.2%	0.80 [0.49, 1.31]
testicular germ cell tumor (n=156)	-0.24143482	0.46044879	2.4%	0.79 [0.32, 1.94]
thyroid carcinoma (n=572)	0.13627762	0.38644685	3.5%	1.15 [0.54, 2.44]
**Total (95% CI)**			**100.0%**	**1.05 [0.91, 1.21]**

Heterogeneity: Chi² = 15.07, df = 19 (P = 0.72); I² = 0%

Test for overall effect: Z = 0.71 (P = 0.48)

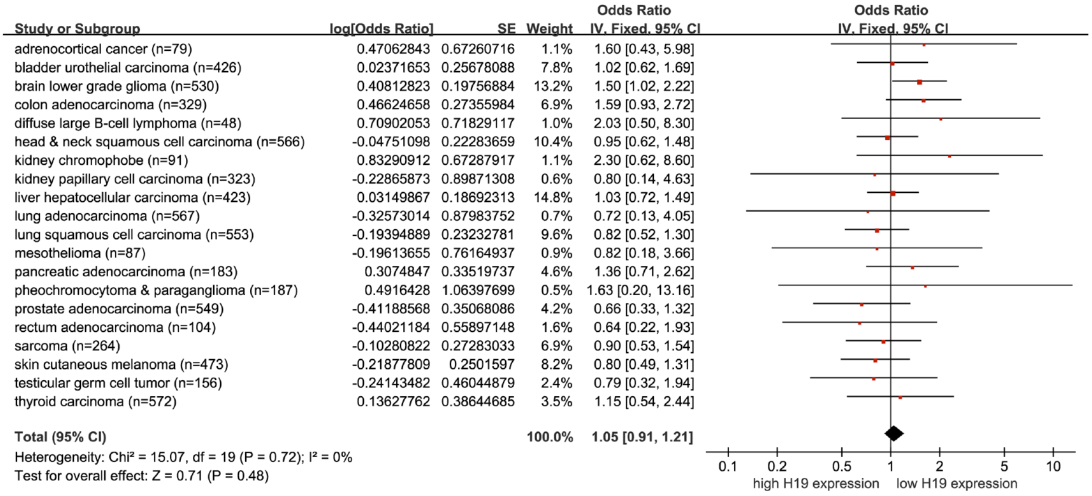

Supplementary Figure S4: Pooling analysis estimate for RFS of H19 expression in non-female cancer patients from the TCGA dataset. The relapse-free survival (RFS) of H19 expression in non-female cancer patients from the Pan-Cancer cohort of the TCGA database. The size of the blocks or diamonds represents the weight for the random-effect model in the meta-analysis. HR > 1 indicates that high H19 expression is correlated with a more unfavorable RFS.

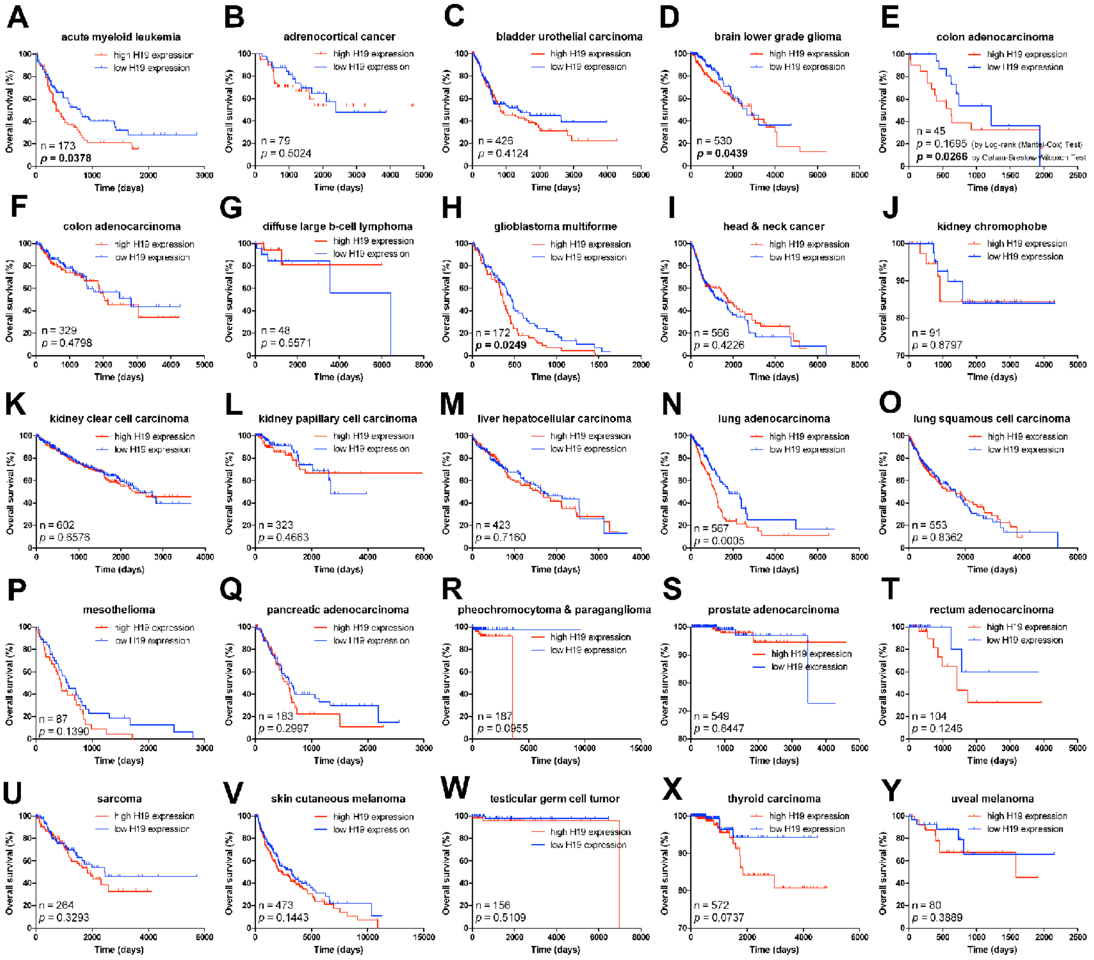

Supplementary Figure S5: Kaplan-Meier estimate for OS of high H19 expression in non-female cancer patients. The overall survival (OS) of high H19 expression in acute myeloid leukemia **A.** adrenocortical cancer **B.** bladder urothelial carcinoma **C.** brain lower grade glioma **D.** cholangiocarcinoma **E.** colon adenocarcinoma **F.** diffuse large B-cell lymphoma **G.** glioblastoma multiforme **H.** head & neck squamous cell carcinoma **I.** kidney chromophobe **J.** kidney clear cell carcinoma **K.** kidney papillary cell carcinoma **L.** liver hepatocellular carcinoma **M.** lung adenocarcinoma **N.** lung squamous cell carcinoma **O.** mesothelioma **P.** pancreatic adenocarcinoma **Q.** pheochromocytoma & paraganglioma **R.** prostate adenocarcinoma **S.** rectum adenocarcinoma **T.** sarcoma **U.** skin cutaneous melanoma **V.** testicular germ cell tumor **W.** thyroid carcinoma **X.** and uveal melanoma **Y.** patients from TCGA datasets.

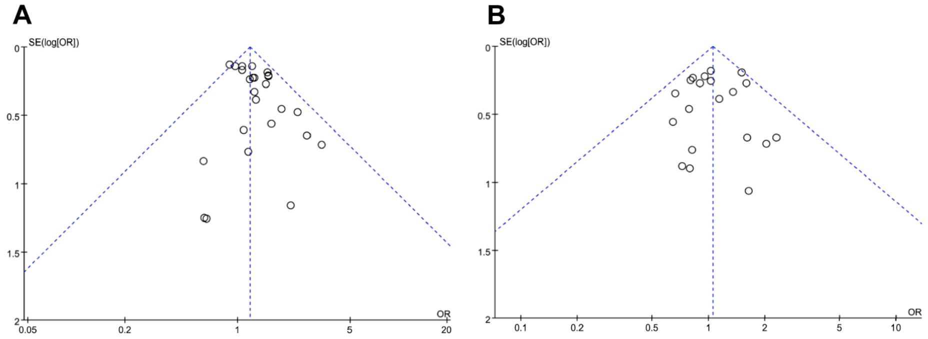

Supplementary Figure S6: Funnel plots on HRs of H19 expression in non-female cancer patients from the TCGA. Funnel plots illustrating significant symmetry on HR for overall survival (OS) **A.** and relapse-free survival (RFS) **B.** in non-female cancer patients from the Pan-Cancer cohort of TCGA database.

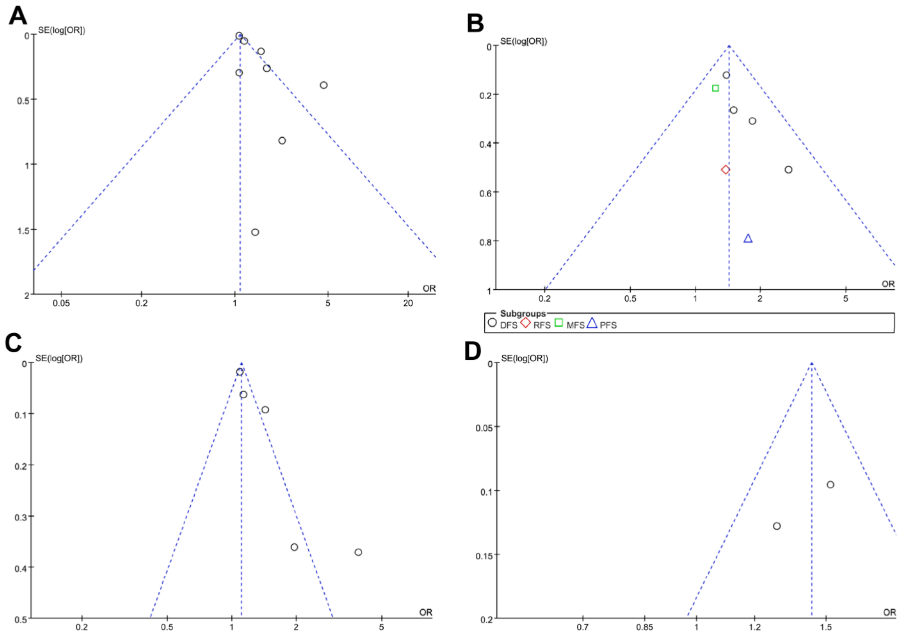

Supplementary Figure S7: Funnel plots on HRs of H19 expression in non-female cancer patients from the available literature. Funnel plots illustrating limited symmetry on HR for overall survival (OS) **A.** and significant asymmetry on HR for disease-free survival (DFS)/relapse-free survival (RFS)/metastasis-free survival (MFS)/ progression-free survival (PFS) **B.** from univariate analysis in non-female cancer patients. Funnel plots illustrated a limited symmetry on HR for OS **C.** and significant asymmetry on HR for DFS **D.** from multivariate analysis in non-female cancer patients.

Supplementary Table S1: The clinic-pathological characteristics of 199 uterine corpus endometrioid carcinoma patients according to H19 expression.

See Supplementary File 1

Supplementary Table S2: The clinic-pathological characteristics of 308 cervical & endocervical adenocarcinoma patients according to H19 expression.

See Supplementary File 2

Supplementary Table S3: The clinic-pathological characteristics of 57 uterine carcinosarcoma patients according to H19 expression.

See Supplementary File 3

Supplementary Table S4: The clinic-pathological characteristics of 1215 breast cancer patients according to H19 expression.

See Supplementary File 4

Supplementary Table S5: The clinic-pathological characteristics of 266 ovarian cancer patients according to H19 expression.

See Supplementary File 5

Supplementary Table S6: The clinic-pathological characteristics of 419 ovarian cancer patients according to H19 expression.

See Supplementary File 6
