# Supplementary material for: High lncRNA H19 expression as prognostic indicator: data mining in female cancers and polling analysis in non-female cancers
Source: Oncotarget. 2016 Dec 1;8(1):1655–67. doi: 10.18632/oncotarget.13768 (PMC5352086; doi:10.18632/oncotarget.13768)
Supplement: Supplementary file 2 [file oncotarget-08-1655-s002.docx]

**Table S1: The clinic-pathological characteristics of 199 uterine corpus endometrioid carcinoma patients according to H19 expression**

| Characteristic | Total | H19 expression | | *p* |
| --- | --- | --- | --- | --- |
|  |  | high | low |  |
| No. of patients | 199 | 50 | 149 | — |
| Gender |  |  |  | — |
| Female | 177 | 41(23.16%) | 136 (76.84%) |  |
| Male | 0 | 0 | 0 |  |
| Age |  |  |  | 0.869 |
| <60 | 50 | 12(24.00%) | 38(76.00%) |  |
| ≥60 | 127 | 29(22.83%) | 98(77.17%) |  |
| Clinical stage |  |  |  | 0.017 |
| I | 97 | 16(16.49%) | 81(83.51%) |  |
| II | 25 | 4(16.00%) | 21(84.00%) |  |
| III | 45 | 17(37.78%) | 28(62.22%) |  |
| IV | 10 | 4(4.00%) | 6(60.00%) |  |
| X | 0 | 0 | 0 |  |
| Grade |  |  |  | 0.073 |
| G1 | 13 | 3(23.08%) | 10(76.82%) |  |
| G2 | 20 | 1(5.00%) | 19(95.00%) |  |
| G3 | 140 | 35(25.00%) | 105(75.00%) |  |
| G4 | 0 | 0 | 0 |  |
| High grade | 4 | 2(50.00%) | 2(50.00%) |  |
| Sample type |  |  |  | 0.223 |
| Solid Tissue Normal | 23 | 9(39.13%) | 14(60.87%) |  |
| Primary Tumor | 175 | 41(23.43%) | 134(76.57%) |  |
| Metastatic | 0 | 0 | 0 |  |
| Recurrent Tumor | 1 | 0(0.00%) | 1(100.00%) |  |
| Weight |  |  |  | 0.267 |
| ≤80 | 83 | 23(27.71%) | 60(72.29%) |  |
| >80 | 79 | 16(20.25%) | 63(79.75%) |  |
| Height |  |  |  | 0.751 |
| ≤161 | 82 | 19(23.17%) | 63(76.83%) |  |
| >161 | 79 | 20(25.32%) | 59(74.68%) |  |
| BMI |  |  |  | 0.278 |
| <25 | 151 | 38(25.17%) | 113(74.83%) |  |
| ≥25 | 10 | 1(10.00%) | 9(90.00%) |  |
| Colorectal cancer |  |  |  | 0.584 |
| positive | 1 | 0(0.00%) | 1(100.00%) |  |
| Negative | 130 | 30(23.08%) | 100(76.92%) |  |
| Diabetes |  |  |  | 0.325 |
| Yes | 32 | 6(17.85%) | 26(81.25%) |  |
| No | 87 | 24(27.00%) | 63(72.41%) |  |
| hypertension |  |  |  | 0.021 |
| Yes | 86 | 15(17.44%) | 71(82.56%) |  |
| No | 45 | 16(35.56%) | 29(64.44%) |  |
| Pregnancies |  |  |  | 0.992 |
| 0 | 15 | 4(26.67%) | 11(73.33%) |  |
| 1 | 11 | 3(27.27%) | 8(72.73%) |  |
| 2 | 41 | 9(21.85%) | 32(78.05%) |  |
| 3 | 26 | 7(26.93%) | 19(73.08%) |  |
| 4+ | 31 | 7(22.58%) | 24(77.42%) |  |
| Anatomic neoplasm subdivision |  |  |  | — |
| Endometrium | 199 | 50 | 149 |  |
| Myometrium | 0 | 0 | 0 |  |
| Fundus uteri | 0 | 0 | 0 |  |
| Lower uterine segment/ Isthmus uteri | 0 | 0 | 0 |  |
| Histological type |  |  |  | <0.0001 |
| Endometrioid endometrial adenocarcinoma | 106 | 12(11.32%) | 94(88.68%) |  |
| Mixed serous and endometrioid | 11 | 7(63.64%) | 4(36.36%) |  |
| Serous endometrial adenocarcinoma | 60 | 22(36.67%) | 38(63.33%) |  |
| Menopause status |  |  |  | 0.641 |
| Post | 145 | 36(24.83%) | 109(75.17%) |  |
| Peri | 2 | 0(0.00%) | 2(100.00%) |  |
| Pre | 11 | 2(18.18%) | 9(81.82%) |  |
