# Supplementary material for: High lncRNA H19 expression as prognostic indicator: data mining in female cancers and polling analysis in non-female cancers
Source: Oncotarget. 2016 Dec 1;8(1):1655–67. doi: 10.18632/oncotarget.13768 (PMC5352086; doi:10.18632/oncotarget.13768)
Supplement: Supplementary file 3 [file oncotarget-08-1655-s003.docx]

**Table S2: The clinic-pathological characteristics of 308 cervical & endocervical adenocarcinoma patients according to H19 expression**

| Characteristic | Total | H19 expression | | *p* |
| --- | --- | --- | --- | --- |
|  |  | high | low |  |
| No. of patients | 308 | 154 | 154 | — |
| Gender |  |  |  | — |
| Female | 305 | 154(50.49%) | 151(49.51%) |  |
| Male | 0 | 0 | 0 |  |
| Age |  |  |  | 0.104 |
| <60 | 250 | 127(52.92%) | 113(47.08%) |  |
| ≥60 | 65 | 27(41.54%) | 38(58.46%) |  |
| Clinical stage |  |  |  | 0.867 |
| I | 160 | 82(51.25%) | 78(48.75%) |  |
| II | 71 | 38(53.52%) | 33(46.48%) |  |
| III | 46 | 21(45.65%) | 25(54.35%) |  |
| IV | 21 | 11(52.38%) | 10(47.62%) |  |
| T (Tumor) |  |  |  | 0.469 |
| Tis | 1 | 1(100%) | 0(0%) |  |
| 1 | 139 | 70(50.36%) | 69(49.64%) |  |
| 2 | 73 | 36(49.32%) | 37(50.68%) |  |
| 3 | 21 | 13(61.90%) | 8(38.10%) |  |
| 4 | 9 | 2(22.22%) | 7(77.78%) |  |
| X | 17 | 7(41.18%) | 10(58.82%) |  |
| N (Node) |  |  |  | 0.829 |
| 0 | 135 | 70(51.85%) | 65(48.15%) |  |
| 1 | 60 | 27(45.00%) | 33(55.00%) |  |
| X | 65 | 32(49.23%) | 33(50.77%) |  |
| M (Metastasis) |  |  |  | 0.257 |
| 0 | 115 | 54(46.96%) | 61(53.04%) |  |
| 1 | 10 | 8(80.00%) | 2(20.00%) |  |
| X | 130 | 65(50.00%) | 65(50.00%) |  |
| Grade |  |  |  | 0.208 |
| G1 | 20 | 10(50.00%) | 10(50.00%) |  |
| G2 | 136 | 63(46.32%) | 73(53.68%) |  |
| G3 | 118 | 68(57.63%) | 50(42.37%) |  |
| G4 | 1 | 1(100%) | 0(0%) |  |
| GX | 23 | 9(39.13%) | 14(60.87%) |  |
| Sample type |  |  |  | 0.311 |
| Solid Tissue Normal | 3 | 2(66.67%) | 1(33.33%) |  |
| Primary Tumor | 303 | 152(50.17%) | 151(49.83%) |  |
| Metastatic | 2 | 0(0%) | 2(100%) |  |
| Recurrent Tumor | 0 | 0 | 0 |  |
| Weight |  |  |  | 0.854 |
| ≤71 | 138 | 71(41.18%) | 67(48.55%) |  |
| >71 | 137 | 72(52.55%) | 65(47.45%) |  |
| Height |  |  |  | 0.047 |
| ≤161 | 135 | 61(45.19%) | 74(54.81%) |  |
| >161 | 127 | 73(57.48%) | 54(42.52%) |  |
| BMI |  |  |  | 0.169 |
| <25 | 256 | 131(51.17%) | 125(48.83%) |  |
| ≥25 | 2 | 2(100.00%) | 0(0%) |  |
| Pregnancies |  |  |  | 0.156 |
| 0 | 16 | 9(56.25%) | 7(43.75%) |  |
| 1 | 30 | 17(56.67%) | 13(43.33%) |  |
| 2 | 47 | 28(59.57%) | 19(40.43%) |  |
| 3 | 50 | 21(42.00%) | 29(58.00%) |  |
| 4+ | 122 | 64(52.46%) | 58(47.54%) |  |
| Ectopic pregnancies |  |  |  | 0.542 |
| 0 | 105 | 57(54.29%) | 48(45.71%) |  |
| 1 | 10 | 5(50.00%) | 5(50.00%) |  |
| 2 | 1 | 0(0%) | 1(100%) |  |
| Breast feeding |  |  |  | — |
| Yes | 0 | 0 | 0 |  |
| No | 74 | 40(54.05%) | 34(45.95%) |  |
| Pregnancy spontaneous abortion |  |  |  | 0.297 |
| 0 | 92 | 53(57.61%) | 39(42.39%) |  |
| 1 | 43 | 21(48.84%) | 22(51.16%) |  |
| 2 | 7 | 4(57.14%) | 3(42.86%) |  |
| 3 | 1 | 1(100%) | 0(0%) |  |
| 4+ | 4 | 3(75.00%) | 1(25.00%) |  |
| Tobacco smoking history |  |  |  | 0.488 |
| Lifelong non- smoker | 143 | 82(57.34%) | 61(42.66%) |  |
| Current smoker | 67 | 30(44.78%) | 37(55.22%) |  |
| Current reformed smoker for ≤ 15 years | 39 | 20(51.28%) | 19(48.72%) |  |
| Current reformed smoker for > 15 years | 9 | 4(44.44%) | 5(55.56%) |  |
| Current Reformed Smoker, Duration Not Specified | 3 | 2(66.67%) | 1(33.33%) |  |
| Histological type |  |  |  | 0.977 |
| Adenosquamous | 5 | 3(60.00%) | 2(40.00%) |  |
| Cervical Squamous Cell Carcinoma | 252 | 123(48.81%) | 129(51.19%) |  |
| Endocervical Adenocarcinoma of the Usual Type | 6 | 3(50.00%) | 3(50.00%) |  |
| Lower uterine segment/ Isthmus uteri | 0 | 0 | 0 |  |
| Endocervical Type of Adenocarcinoma | 23 | 13(56.52%) | 10(43.48%) |  |
| Endometrioid Adenocarcinoma of Endocervix | 4 | 2(50.00%) | 2(50.00%) |  |
| Mucinous Adenocarcinoma of Endocervical Type | 15 | 8(53.33%) | 7(46.67%) |  |
| HPV |  |  |  | 0.420 |
| HPV16 | 9 | 3(33.33%) | 6(66.67%) |  |
| HPV18 | 3 | 2(66.67%) | 1(33.33%) |  |
| Other HPV type(s) | 10 | 6(60.00%) | 4(40.00%) |  |
| Menopause status |  |  |  | 0.072 |
| Post | 83 | 40(48.19%) | 43(51.81%) |  |
| Peri | 25 | 9(36.00%) | 16(64.00%) |  |
| Pre | 125 | 72(57.60%) | 53(42.40%) |  |
| Indeterminate | 2 | 2(100.00%) | 0(0.00%) |  |
