# Supplementary material for: High lncRNA H19 expression as prognostic indicator: data mining in female cancers and polling analysis in non-female cancers
Source: Oncotarget. 2016 Dec 1;8(1):1655–67. doi: 10.18632/oncotarget.13768 (PMC5352086; doi:10.18632/oncotarget.13768)
Supplement: Supplementary file 4 [file oncotarget-08-1655-s004.docx]

**Table S3: The clinic-pathological characteristics of 57 uterine carcinosarcoma patients according to H19 expression**

| Characteristic | Total | High H19 expression | Low H19 expression | *p* |
| --- | --- | --- | --- | --- |
| No. of patients | 57 | 28 | 29 |  |
| Sex |  |  |  | — |
| Female | 57 | 28(49.12%) | 29(50.88%) |  |
| Male | 0 | 0 | 0 |  |
| Age |  |  |  | 0.413 |
| <60 | 6 | 2(33.33%) | 4(66.67%) |  |
| ≥60 | 51 | 26(50.98%) | 25(49.02%) |  |
| Clinical stage |  |  |  | 0.905 |
| I | 22 | 10(45.45%) | 12(54.55%) |  |
| II | 5 | 2(40.00%) | 3(60.00%) |  |
| III | 20 | 11(55.00%) | 9(45%) |  |
| IV | 10 | 5(50.00%) | 5(50.00%) |  |
| X | 0 | 0 | 0 |  |
| Sample type |  |  |  | — |
| Solid Tissue Normal | 0 | 0 | 0 |  |
| Primary Tumor | 57 | 28(49.12%) | 29(50.88%) |  |
| Metastatic | 0 | 0 | 0 |  |
| Recurrent Tumor | 0 | 0 | 0 |  |
| Weight |  |  |  | 0.158 |
| ≤72 | 27 | 15(55.56%) | 12(44.44%) |  |
| >72 | 25 | 9(36.00%) | 16(64.00%) |  |
| Height |  |  |  | 0.578 |
| <157.5 | 26 | 13(50.00%) | 13(50.00%) |  |
| >157.5 | 26 | 11(42.31%) | 15(57.69%) |  |
| BMI |  |  |  | 0.119 |
| <25 | 50 | 22(44.00%) | 28(56.00%) |  |
| ≥25 | 2 | 2(100.00%) | 0(0%) |  |
| Colorectal cancer |  |  |  | 0.588 |
| Yes | 1 | 1(100%) | 0(0%) |  |
| No | 50 | 24(48.00%) | 26(52.00%) |  |
| Diabetes |  |  |  | 0.172 |
| Yes | 6 | 2(33.33%) | 4(66.67%) |  |
| No | 45 | 21(46.67%) | 24(53.33%) |  |
| hypertension |  |  |  | 0.850 |
| Yes | 28 | 13(46.43%) | 15(53.57%) |  |
| No | 24 | 12(50.00%) | 12(50.00%) |  |
| Pregnancies |  |  |  | 0.094 |
| 0 | 5 | 0(0%) | 5(100%) |  |
| 1 | 4 | 4(100%) | 0(0%) |  |
| 2 | 19 | 9(47.37%) | 10(52.63%) |  |
| 3 | 15 | 7(46.67%) | 8(53.33%) |  |
| 4+ | 7 | 4(57.14%) | 3(42.86%) |  |
| Anatomic neoplasm subdivision |  |  |  | 0.508 |
| Endometrium | 33 | 15(45.45%) | 18(54.55%) |  |
| Myometrium | 2 | 2(100%) | 0(0%) |  |
| Fundus uteri | 1 | 1(100%) | 0(0%) |  |
| Lower uterine segment/ Isthmus uteri | 2 | 1(50.00%) | 1(50.00%) |  |
| Histological type |  |  |  | 0.065 |
| Homologous | 13 | 10(76.92%) | 3(23.08%) |  |
| Heterologous | 20 | 9(45.00%) | 11(55.00%) |  |
| Malignant Mixed Mullerian Tumor | 24 | 9(37.50%) | 15(62.50%) |  |
| Menopause status |  |  |  | 0.225 |
| Post | 54 | 27(50.00%) | 27(50.00%) |  |
| Peri | 1 | 1(100%) | 0(0%) |  |
