# Supplementary material for: High lncRNA H19 expression as prognostic indicator: data mining in female cancers and polling analysis in non-female cancers
Source: Oncotarget. 2016 Dec 1;8(1):1655–67. doi: 10.18632/oncotarget.13768 (PMC5352086; doi:10.18632/oncotarget.13768)
Supplement: Supplementary file 5 [file oncotarget-08-1655-s005.docx]

Table S4: The clinic-pathological characteristics of 1215 breast cancer patients according to H19 expression

| Characteristic | Total | High H19 expression | Low H19 expression | *p* |
| --- | --- | --- | --- | --- |
| No. of patients | 1215 | 608 | 607 |  |
| Sex |  |  |  | 0.002 |
| Female | 1184 | 582(49.16%) | 602 (50.84%) |  |
| Male | 12 | 11(91.67%) | 1(8.33%) |  |
| Age |  |  |  | 0.889 |
| <60 | 647 | 322(49.77%) | 325(50.23%) |  |
| ≥60 | 549 | 271(49.36%) | 278(50.64%) |  |
| Clinical stage |  |  |  | 0.034 |
| Tis | 1 | 1 | 0 |  |
| I | 204 | 102(50.00%) | 102(50.00%) |  |
| II | 679 | 319(46.98%) | 360(53.02%) |  |
| III | 271 | 150(55.35) | 121(44.65%) |  |
| IV | 21 | 8(38.10%) | 13(61.90%) |  |
| X | 18 | 12(66.67%) | 6(33.33%) |  |
| T (Tumor) |  |  |  | 0.032 |
| 1 | 311 | 163(52.41%) | 148(47.59%) |  |
| 2 | 691 | 32146.45% | 370(53.55%) |  |
| 3 | 143 | 83(58.04%) | 60(41.96%) |  |
| 4 | 47 | 24(51.06%) | 23(48.94%) |  |
| X | 4 | 2(50.00%) | 2(50.00%) |  |
| N |  |  |  | 0.172 |
| 0 | 553 | 263(47.56%) | 290(52.44%) |  |
| 1 | 510 | 206(50.24%) | 204(49.76%) |  |
| 2 | 129 | 65(50.39%) | 64(49.61%) |  |
| 3 | 81 | 47(58.02%) | 34(41.98%) |  |
| X | 23 | 12(52.17%) | 11(47.83%) |  |
| M |  |  |  | 0.018 |
| 0 | 1010 | 489(48.42%) | 521(51.58%) |  |
| 1 | 22 | 9(40.91%) | 13(59.09%) |  |
| X | 164 | 95(57.93) | 69(42.07%) |  |
| Position |  |  |  | 0.006 |
| Left | 625 | 289(46.24%) | 336(53.76%) |  |
| Right | 571 | 304(53.24%) | 267(46.76%) |  |
| Sample type |  |  |  | 0.386 |
| Solid Tissue Normal | 113 | 61(53.98%) | 52 (46.02%) |  |
| Primary Tumor | 1095 | 544(49.68%) | 551 (50.32%) |  |
| Metastatic | 7 | 2(28.57%) | 5(71.43%) |  |
| Recurrent Tumor | 0 | 0 | 0 |  |
| ER |  |  |  | 0.091 |
| Positive | 878 | 453(51.59%) | 425(48.41%) |  |
| Negative | 255 | 111(43.53%) | 144(56.47%) |  |
| PR |  |  |  | 0.068 |
| Positive | 762 | 397(52.10%) | 365(47.90%) |  |
| Negative | 368 | 163(44.29%) | 205(55.71%) |  |
| HER2 |  |  |  | 0.061 |
| Positive | 189 | 100(52.91%) | 89(47.09%) |  |
| Negative | 940 | 454(48.30%) | 486(51.70%) |  |
| Margin status |  |  |  | 0.126 |
| Positive | 82 | 50(60.98%) | 32(39.02%) |  |
| Negative | 998 | 494(49.50%) | 504(50.50%) |  |
