# Supplementary material for: High lncRNA H19 expression as prognostic indicator: data mining in female cancers and polling analysis in non-female cancers
Source: Oncotarget. 2016 Dec 1;8(1):1655–67. doi: 10.18632/oncotarget.13768 (PMC5352086; doi:10.18632/oncotarget.13768)
Supplement: Supplementary file 6 [file oncotarget-08-1655-s006.docx]

**Table S5: The clinic-pathological characteristics of 266 ovarian cancer patients according to H19 expression**

| Characteristic | Total | High H19 expression (%) | Low H19 expression (%) | *p* |
| --- | --- | --- | --- | --- |
| No. of patients | 266 | 133 (50%) | 133 (50%) |  |
| Sex |  |  |  | — |
| Female | 266 | 133 (50%) | 133 (50%) |  |
| Male | 0 | 0 | 0 |  |
| Age |  |  |  | 0.108 |
| <60 | 149 | 68(45.64%) | 81 (54.36%) |  |
| ≥60 | 117 | 65(55.56%) | 52 (44.44%) |  |
| Clinical stage |  |  |  | 0.374 |
| I | 0 | 0 | 0 |  |
| II | 19 | 12(63.16%) | 7(36.84%) |  |
| III | 213 | 102(47.89%) | 111(52.11%) |  |
| IV | 33 | 18(54.55%) | 15(45.45%) |  |
| Grade |  |  |  | 0.289 |
| G1 | 1 | 1(100%) | 0 |  |
| G2 | 33 | 20(60.61%) | 13(39.39%) |  |
| G3 | 225 | 107(47.56%) | 118(52.44%) |  |
| G4 | 1 | 1(100%) | 0 |  |
| GB | 1 | 1(100%) | 0 |  |
| GX | 3 | 1(33.33%) | 2(66.67%) |  |
| Sample type |  |  |  | 0.082 |
| Solid Tissue Normal | 0 | 0 | 0 |  |
| Primary Tumor | 263 | 130(49.43%) | 133(50.57%) |  |
| Metastatic | 0 | 0 | 0 |  |
| Recurrent Tumor | 3 | 3(100%) | 0 |  |
| Subdivision |  |  |  | 0.877 |
| Left | 34 | 17(50%) | 17(50%) |  |
| Right | 34 | 19(55.88%) | 15(44.12%) |  |
| Bilateral | 185 | 90(48.65%) | 95(51.35%) |  |
| Tumor residual disease |  |  |  | 0.453 |
| No Macroscopic disease | 54 | 24(44.44%) | 30(55.56%) |  |
| 1-10 mm | 119 | 67(56.30%) | 52(43.70%) |  |
| 11-20 mm | 20 | 9(45.00%) | 11(55.00%) |  |
| >20 mm | 42 | 19(55.88%) | 23(54.76%) |  |
| Lymphatic invasion |  |  |  | 0.328 |
| Positive | 70 | 40(57.14%) | 30(42.68%) |  |
| Negative | 39 | 17(43.59%) | 22(56.41%) |  |
| Venous invasion |  |  |  | 0.782 |
| Positive | 38 | 18(47.37%) | 20(52.63%) |  |
| Negative | 44 | 24(54.55%) | 20(45.45%) |  |
