# Supplementary material for: High lncRNA H19 expression as prognostic indicator: data mining in female cancers and polling analysis in non-female cancers
Source: Oncotarget. 2016 Dec 1;8(1):1655–67. doi: 10.18632/oncotarget.13768 (PMC5352086; doi:10.18632/oncotarget.13768)
Supplement: Supplementary file 7 [file oncotarget-08-1655-s007.docx]

**Table S6: The clinic-pathological characteristics of 419 ovarian cancer patients according to H19 expression**

| Characteristic | Total | High H19 expression | Low H19 expression | *p* |
| --- | --- | --- | --- | --- |
| No. of patients | 419 | 209 (49.88%) | 210(50.12%) |  |
| Sex |  |  |  | — |
| Female | 419 | 209 (49.88%) | 210(50.12%) |  |
| Male | 0 | 0 | 0 |  |
| Age |  |  |  | 0.010 |
| <60 | 223 | 98(43.95%) | 125(56.05%) |  |
| ≥60 | 196 | 111(56.63%) | 85(43.37) |  |
| Clinical stage |  |  |  | 0.743 |
| I | 0 | 0 | 0 |  |
| II | 23 | 13(56.52%) | 10(43.48%) |  |
| III | 331 | 162(48.94%) | 169(51.06%) |  |
| IV | 62 | 32(51.61%) | 30(48.39%) |  |
| Grade |  |  |  | 0.170 |
| G1 | 1 | 0(0%) | 1(100%) |  |
| G2 | 52 | 31(59.62%) | 21(40.38%) |  |
| G3 | 357 | 173(48.46%) | 184(51.54%) |  |
| G4 | 1 | 1(100%) | 0(0%) |  |
| GB | 1 | 1(100%) | 0(0%) |  |
| GX | 5 | 1(20.00%) | 4(80.00%) |  |
| Sample type |  |  |  | 0.698 |
| Solid Tissue Normal | 0 | 0 | 0 |  |
| Primary Tumor | 412 | 205(49.76%) | 207(50.24%) |  |
| Metastatic | 0 | 0 | 0 |  |
| Recurrent Tumor | 7 | 4(57.14%) | 3(42.86%) |  |
| Position |  |  |  | 0.762 |
| Left | 56 | 26(46.43%) | 30(53.57%) |  |
| Right | 46 | 26(56.52%) | 20(43.48%) |  |
| Bilateral | 292 | 144(49.32%) | 148(50.68%) |  |
| Tumor residual disease |  |  |  | 0.858 |
| No Macroscopic disease | 76 | 38(50.00%) | 38(50.00%) |  |
| 1-10 mm | 196 | 104(53.06%) | 92(46.94%) |  |
| 11-20 mm | 29 | 15(51.72%) | 14(48.28%) |  |
| >20 mm | 76 | 36(47.37%) | 40(52.63%) |  |
| Lymphatic invasion |  |  |  | 0.127 |
| Positive | 55 | 23(41.82%) | 32(58.18%) |  |
| Negative | 106 | 61(57.55%) | 45(42.45%) |  |
| Venous invasion |  |  |  | 0.791 |
| Positive | 48 | 22(45.83%) | 26(54.17%) |  |
| Negative | 65 | 34(52.31%) | 31(47.69%) |  |
